# Celecoxib normalizes the tumor microenvironment and enhances small nanotherapeutics delivery to A549 tumors in nude mice

**DOI:** 10.1038/s41598-017-09520-7

**Published:** 2017-08-30

**Authors:** Bo Zhang, Kai Jin, Ting Jiang, Lanting Wang, Shun Shen, Zimiao Luo, Yanyan Tuo, Xianping Liu, Yu Hu, Zhiqing Pang

**Affiliations:** 1 School of Pharmacy, Fudan University, Key Laboratory of Smart Drug Delivery, Ministry of Education, 826 Zhangheng Road, Shanghai, 201203 PR China; 2 Institute of Hematology, Union Hospital, Tongji Medical College, Huazhong University of Science and Technology, Wuhan, Hubei 430022 PR China; 3 Department of Radiology, Huashan Hospital, Fudan University, 12 Wulumuqi Middle Road, Shanghai, 200040 PR China

## Abstract

Barriers presented by the tumor microenvironment including the abnormal tumor vasculature and interstitial matrix invariably lead to heterogeneous distribution of nanotherapeutics. Inspired by the close association between cyclooxygenase-2 (COX-2) and tumor-associated angiogenesis, as well as tumor matrix formation, we proposed that tumor microenvironment normalization by COX-2 inhibitors might improve the distribution and efficacy of nanotherapeutics for solid tumors. The present study represents the first time that celecoxib, a special COX-2 inhibitor widely used in clinics, was explored to normalize the tumor microenvironment and to improve tumor nanotherapeutics delivery using a human-derived A549 tumor xenograft as the solid tumor model. Immunofluorescence staining of tumor slices demonstrated that oral celecoxib treatment at a dose of 200 mg/kg for two weeks successfully normalized the tumor microenvironment, including tumor-associated fibroblast reduction, fibronectin bundle disruption, tumor vessel normalization, and tumor perfusion improvement. Furthermore, it also significantly enhanced the *in vivo* accumulation and deep penetration of 22-nm micelles rather than 100-nm nanoparticles in tumor tissues by *in vivo* imaging and distribution experiments and improved the therapeutic efficacy of paclitaxel-loaded micelles in tumor xenograft-bearing mouse models in the pharmacodynamics experiment. As celecoxib is widely and safely used in clinics, our findings may have great potential in clinics to improve solid tumor treatment.

## Introduction

**Figure 1:**
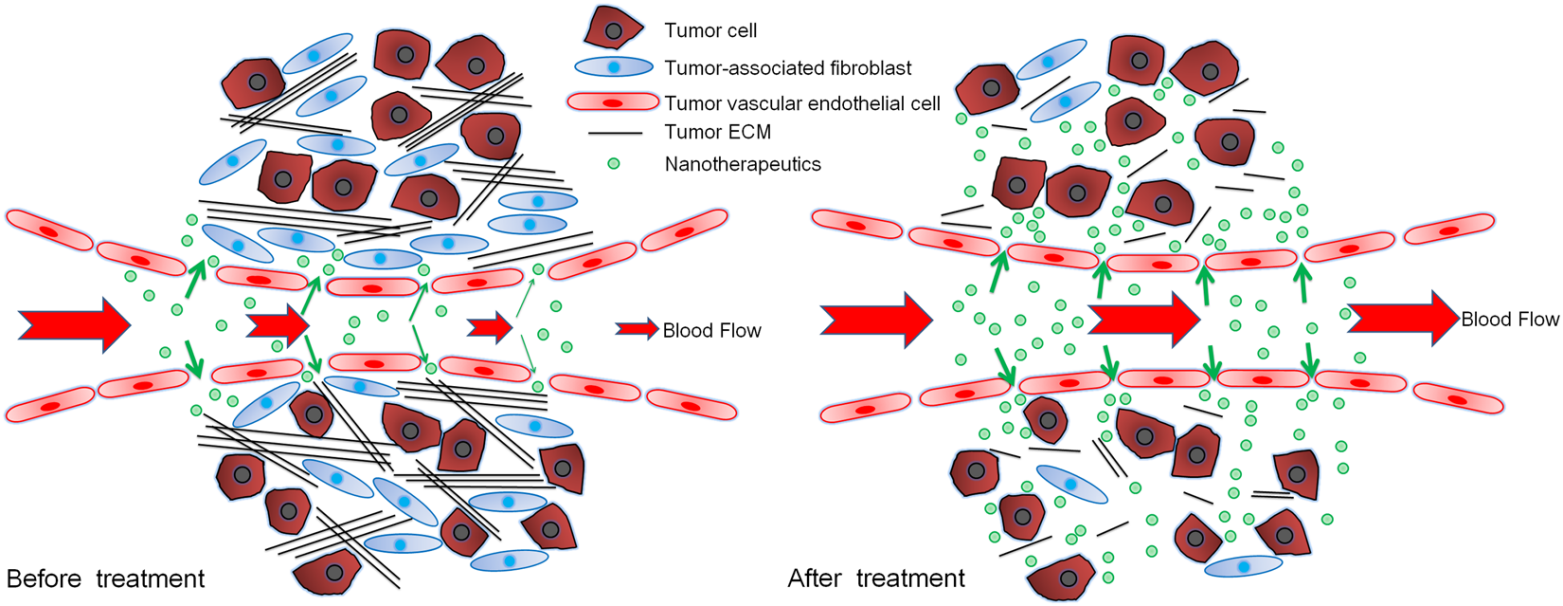
Schematic graphs of the tumor microenvironment and nanotherapeutics delivery to tumors rich in vessels and ECM before and after celecoxib treatment. Before celecoxib treatment, the tumor vessels were leaky and compressed by tumor ECM and TAF, which were a main contributor to the heterogeneous perfusion in tumors and, accordingly, the compromised nanotherapeutics delivery to tumors. As a comparison, celecoxib treatment reduced TAF, disrupted tumor ECM, and repaired tumor vessels to enhance their maturity, which ultimately improved tumor perfusion and enhanced tumor nanotherapeutics delivery.

Nanotherapeutics was developed to improve the therapeutic benefit of drugs to solid tumors by achieving a high accumulation of drugs in tumor tissues while sparing in normal tissues^1, 2^. However, barriers presented by the tumor microenvironment, including abnormal tumor vasculature, abundant extracellular matrix (ECM), and different types of stroma cells invariably lead to heterogeneous distribution of nanotherapeutics in tumors^3, 4^. The abnormal tumor vessels result in heterogeneous tumor perfusion and extravasation^5^. As the most principal stroma cells, tumor-associated fibroblasts (TAFs) are responsible for tumor ECM synthesis. TAFs and tumor matrix not only compress tumor vessels to decrease tumor perfusion^6^, but also block free access of nanotherapeutics to tumor cells (Fig. 1A)^7, 8^. Consequently, the complex tumor microenvironment compromises the clinical benefits of some anti-tumor nanotherapeutics, such as Doxil and Abraxane, two nanotherapeutics approved by the Food and Drug Administration (FDA) for use in solid tumors^4, 9, 10^. Accordingly, strategies for regulating the tumor microenvironment to uniformly deliver nanotherapeutics throughout tumor tissues with sufficient concentration were urgently needed.

To date, various strategies aimed at improving the accumulation and distribution of nanotherapeutics in tumors have been presented to modify the tumor microenvironment. These strategies include tumor ECM disruption by ECM disruptors, such as matrix metalloproteinases^11^, hyaluronidase^12^, recombinant tissue plasminogen activator^13^, and tumor vessel normalization by vascular endothelial growth factor (VEGF) antibody bevacizumab^14^ and tyrosine kinase inhibitor (TKI) cediranib^15, 16^. However, on the one hand, ECM disruption only re-opened collapsed tumor vessels but exerted no effect on their leaky structure. On the other hand, tumor vessel normalization could not help perfuse those vessels that were still compressed by ECM or TAF^8^. Additionally, the application of those agents in clinics is also limited due to safety concerns, high price, instability, and/or inconsistent therapeutic benefits. Therefore, therapeutic agents possessing a comprehensive capacity to normalize the tumor microenvironment with a favorable cost performance and high potential of clinical translation are still imperative.

Cyclooxygenase-2 (COX-2) is an isoform of COX and is highly expressed at tumor sites^17^. COX-2 and its downstream signal moiety prostaglandin are widely known to be actively involved in tumor associated angiogenesis^17, 18^ and contribute much to tumor ECM formation^19^, indicating that COX-2 might be a favorable target for tumor microenvironment modification. Celecoxib, a special COX-2 inhibitor with no COX-1-associated adverse effects, such as the promotion of bleeding and the induction of gastrointestinal complications, is currently widely used in clinics^20^. Inspired by the close association between COX-2 and tumor-associated angiogenesis as well as tumor ECM formation, we proposed that celecoxib might safely and comprehensively normalize the tumor microenvironment and improve the distribution and efficacy of nanotherapeutics for solid tumors (Fig. 1). Although it has been documented that celecoxib^19, 21^ or celecoxib-loaded nanoparticles^22^ could be utilized to suppress tumor growth or metastasis by inhibition of the COX-2-related signaling pathway, there have been very few studies using COX-2 inhibitors to regulate the tumor microenvironment and help increase the delivery of nanotherapeutics for tumors.

The present study represents the first time wherein celecoxib was explored to modulate the tumor environment and to improve tumor nanotherapeutics delivery, using human-derived lung cancer A549 tumors rich in vessels and ECM as the tumor models^13^ and paclitaxel (PTX)-loaded polyethylene glycol-polylactide (PEG-PLA) micelles as the model nanotherapeutics. The effects of celecoxib on the tumor microenvironment, including tumor vessel normalization, tumor ECM disruption, tumor-associated fibroblast reduction, and tumor perfusion improvement were investigated by immunofluorescence staining. The concomitant effect of celecoxib on the behaviors of nanotherapeutics of different sizes within tumors was also evaluated by *in vivo* imaging and distribution experiments. Finally, a pharmacodynamics experiment was performed to assess the growth inhibition effect of nanotherapeutics combined with celecoxib on A549 tumor xenografts. To the best of our knowledge, this was the first time that COX-2 inhibitors were explored to comprehensively normalize the tumor microenvironment and improve tumor drug delivery. As the tumor microenvironment modulator celecoxib is now widely and safely used in clinics, our findings may have great potential for improving tumor treatment.

## Materials and Methods

### Materials

Celecoxib was purchased from Dalian Meilun Biotech Co., Ltd (Dalian, China). Hoechst 33342 were purchased from Beyotime^®^ Biotechnology Co., Ltd. (Nantong, China). DyLight^®^ 488-labeled tomato lectin (Lycopersicon esculentum) (DyLight^®^ 488-labeled lectin) was purchased from Vector (USA). Cy™ 3-conjugated SMA-α mouse monoclonal primary antibody and fluorescence tracker coumarin-6 were from Sigma (USA). 1,1′-Dioctadecyl-3,3,3′,3′-tetramethylindo-tricarbocyanineiodide (DiR), a near-infrared dye, was purchased from Biotium (Invitrogen, USA). FAPα rabbit polyclonal primary antibody and fibronectin rabbit polyclonal primary antibody were obtained from Santa Cruz biotechnology (USA). CD31 goat polyclonal primary antibody was from R&D (USA). Cy™ 3-conjugated Affinipure donkey anti-goat secondary antibody, Alexa Fluor^®^ 647-conjugated rabbit-goat secondary antibody and Alexa Fluor^®^ 488-conjugated donkey-rabbit secondary antibody were obtained from Jackson (USA). MPEG_2000_-PLA_2000_ was purchased from Jinan Daigang Biomaterial Co., Ltd (Jinan, China). Sodium cholate was purchased from Shanghai Chemical (Shanghai, China). Fetal bovine serum (FBS), trypsin-ethylenediaminetetraacetic acid (trypsin-EDTA, 0.25%), cell culture medium, and penicillin-streptomycin were ordered from Gibco (CA). Deionized water supplied from the Millipore Simplicity System (Millipore, Bedford, MA) was used throughout the entire study. All other reagents and chemicals were of analytical grade and were purchased from Sinopharm Chemical Reagent (Shanghai, China).

### Cells and animal models

A549 cell lines were ordered from the Chinese Academy of Sciences Cell Bank (Shanghai, China), authenticated through a short tandem repeat (STR) profiles analysis by Beijing Microread Genetics Co. Ltd, and cultured under recommended conditions. Male Balb/c nude mice (aged six to eight weeks) were bought from the Shanghai SLAC Lab Animal Co., Ltd. (Shanghai, China) and used under the current regulations and standards of the Animal Ethics Committee of Fudan University. To produce tumor xenografts, 100 μl of tumor cell suspension (5 × 10^7^ cells/ml) was injected subcutaneously into nude mice. Tumor volume (*V*) was approximated with a caliper using the formula: $V\,=\,0.5\,\times \,a\times {b}^{2}$, where *a* was the maximum perpendicular diameter, and *b* was the minimum perpendicular diameter. When tumor xenografts reached a diameter of 4 to 5 mm, mouse models were selected for subsequent experiments.

### Celecoxib treatment

Celecoxib powder was grinded with 4% sodium carboxymethylcellulose to obtain a celecoxib suspension with a celecoxib concentration of 20 mg/ml. Blank celecoxib suspension was prepared with the same method, except that no celecoxib was added to the 4% sodium carboxymethylcellulose. All the suspensions were prepared before use. Mouse models were randomly divided into two groups and orally treated with celecoxib suspension at a dose of 200 mg/kg/day or an equal volume of blank celecoxib suspension as a control for two weeks. To identify the potential adverse effects of celecoxib, the tumor size and body weight of the mice in each group (n = 6) were carefully monitored every other day throughout celecoxib treatment. When celecoxib treatment ended, mice were sacrificed and major organs were harvested for frozen slice preparation and hematoxylin-eosin (H&E) staining.

### Histological analysis of the tumor microenvironment normalization by celecoxib treatment

When the two-week celecoxib treatment period ended, tumors were collected after heart perfusion with 4% paraformaldehyde, immersed in 4% paraformaldehyde for 24 h, followed by incubation in 10% sucrose overnight and 30% sucrose overnight in sequence, and then embedded in Optimal Cutting Temperature (OCT) compound for frozen slices of 5-μm thickness. For immunofluorescence staining, tumor slices were blocked with 10% goat serum at room temperature for 1 h, incubated with primary antibody at 4 °C overnight, and then labeled with its corresponding secondary antibody the next day as described elsewhere^23^. TAF was labeled with FAPα rabbit polyclonal primary antibody (1:100 diluted) and Alexa Fluor^®^ 488-conjugated donkey-rabbit secondary antibody (1:100 diluted)^24^. Fibronectin, an important tumor ECM marker^25, 26^, was labeled with fibronectin rabbit polyclonal primary antibody (1:100 diluted) and Alexa Fluor^®^ 488-conjugated donkey-rabbit secondary antibody (1:100 diluted). Vascular endothelial cells were labeled with CD31 goat polyclonal primary antibody (1:100 diluted) and Alexa Fluor^®^ 647-conjugated rabbit-goat secondary antibody (1:200 diluted). Pericytes were labeled with Cy™ 3-conjugated SMA-α mouse monoclonal primary antibody (1:200 diluted). Digital images of tumor slices were captured by confocal microscopy (ZEISS, 710, LSM, Germany). To assess the effect of celecoxib treatment on TAF number in tumor tissues, the percentage of TAF (FAPα+) in tumor tissue cells was calculated in six randomly-assigned regions of each tumor (n = 3) at 120× magnification. To evaluate the fibronectin disruption effect of celecoxib treatment, the fluorescence intensity of fibronectin in tumor slices from six randomly-assigned regions in each tumor (n = 3) was quantified by the ZEN 2012 software at 120× magnification. CD31-positive blood vessels that co-localized with pericytes were generally identified as normalized blood vessels^27, 28^. To demonstrate the effect of celecoxib treatment on tumor vessel normalization, the co-localization of the pericytes and endothelial cells in the tumor sections were captured in six randomly-assigned regions in each tumor (n = 3) at 200× magnification (ZEISS, 710, LSM, Germany) and further analyzed using the ImageJ (National Institutes of Health (NIH)) software.

For tumor perfusion detection, when the two-week celecoxib treatment period ended, mouse models received an intravenous (i.v.) injection of 100 μg of DyLight^®^ 488-lectin. One hour post-injection, mouse models were sacrificed followed by heart-perfusion with 4% paraformaldehyde. Tumors were harvested and sectioned for CD31 staining by CD31 goat polyclonal primary antibody (1:100) as described above. Tumor slices were observed under confocal microscopy (ZEISS, 710, LSM, Germany). The density of microvesssels labeled with CD31 from six randomly-assigned regions in each tumor (n = 3) was calculated by Image J software (NIH) at 120× magnification and expressed in terms of the number of vessels per unit area (mm^2^)^19^. CD31-positive blood vessels co-localized with DyLight^®^ 488-lectin were identified as well perfused blood vessels^27^, and tumor perfusion was indicated as the percentage of well perfused vessels in all tumor vessels. To assess the tumor perfusion, the co-localization of CD31-positive signals and DyLight^®^ 488-lectin signals (perfused vessels) in the tumor sections were captured in six randomly-assigned regions in each tumor (n = 3) at 200× magnification and further analyzed using the ImageJ software (NIH).

### Preparation and characterization of nanotherapeutics

Micelles based on methoxy poly(ethylene glycol)-poly(lactide) copolymer (MPEG-PLA), model nanotherapeutics with small size, were developed by a thin-film hydration method as previously reported^29^. Briefly, 30 mg of MPEG-PLA were dissolved in 5 ml of acetonitrile, and acetonitrile was removed by rotary evaporation for 2 h at 40 °C with a ZX-98 rotary evaporator (Shanghai Institute of Organic Chemistry, China). The thin polymer film that formed in the round-bottom flask was hydrated with 2 ml of water and the micelle solution was obtained. Coumarin-6-, DiR-labeled and paclitaxel (PTX)-loaded micelles were prepared with the same method except, that 30 µg of coumarin-6, 200 µg of DiR or 10 mg of PTX were added into the dissolved polyester materials in advance, and the collected micelles were filtrated with a 0.22-µm filter to remove unloaded drug aggregates. Particle size and zeta potential of blank micelles and PTX-loaded micelles were analyzed by dynamic light scattering (DLS) using a Malvern Nano ZS (Malvern Instruments, UK). The morphological examination of micelles was observed using a transmission electron microscope (TEM) (H-600, Hitachi, Japan) after negative staining with 2% phosphotungstic acid. The drug loading of PTX in micelles was determined by the HPLC method^30^. The *in vitro* release behavior of PTX from Micelles-PTX was evaluated by a dialysis method using phosphate buffered saline (PBS) (0.01 M, pH 7.4) with 0.5% Tween-80 as the release medium as previously described^30^. One hundred-nanometer nanoparticles based on MPEG-PLA, model nanotherapeutics with a larger size, were prepared and characterized as described in the supplementary information.

### *In vivo* imaging

At the end of the two-week celecoxib treatment period, the mouse models were injected with DiR-labeled micelles at the DiR dose of 0.5 mg/kg. Twenty-four hours later, the *in vivo* fluorescence imaging of mouse models in each group (n = 4) was performed with the *In Vivo* IVIS spectrum imaging system (PerkinElmer, USA). The mouse models were then sacrificed, followed by heart perfusion with saline. Tumors and major organs, including livers, spleens, hearts, lungs, kidneys, and brains were collected. The *ex vivo* imaging of tumors and those major organs were performed with the *In Vivo* IVIS spectrum imaging system (PerkinElmer, USA). Additionally, the major organs and tumors were carefully weighed and then homogenized in 0.01 M PBS (pH = 7.4). The fluorescence intensity of each sample was analyzed by a Tecan Infinite M200 Pro Multiplate Reader (Switzerland) with an excitation wavelength of 748 nm and an emission wavelength of 780 nm^31^.

### Micelles distribution in tumors

After the two-week celecoxib treatment period ended, the mouse models in each group (n = 4) were injected with coumarin-6-labeled micelles at the coumarin-6 dose of 0.5 mg/kg. Four hours later, the mouse models were sacrificed, followed by heart perfusion with 4% paraformaldehyde. The tumors were treated and sectioned for CD31 staining as described above. Tumor slices were observed under a confocal imaging microscope (ZEISS, 710, LSM, Germany). The micelle fluorescence intensity of tumor slices from six randomly-assigned regions in each tumor (n = 4) was quantified by the ZEN 2012 software. The penetration profiles of micelles from the nearest blood vessels were also characterized by the ZEN 2012 software.

### Tumor growth inhibition experiment

Twenty-four A549 tumor xenograft-bearing mouse models were randomly divided into 4 groups: the control group, the control plus Micelles-PTX group, the celecoxib group, and the celecoxib plus Micelles-PTX group. For the celecoxib plus Micelles-PTX group or the celecoxib group, celecoxib suspensions were orally administered to the mouse models at a dosage of 200 mg/kg/day for 2 weeks as described above, and then treated with or without Micelles-PTX at the PTX dosage of 6 mg/kg every third day for five times. For the control plus Micelles-PTX group or the control group, equal volumes of blank celecoxib suspensions were orally administered to mouse models daily for two weeks, and then mice were treated with or without Micelles-PTX as described above. The day of Micelles-PTX initiation was recorded as day 0. Tumor size was recorded with a caliper and tumor volumes were approximated every other day for 16 days. The tumor growth curve was drawn to analyze the therapeutic efficacy of different treatments. Sixteen days later, tumor xenografts were resected and imaged, and their wet weight was measured. Then, tumor xenografts were fixed with 4% paraformaldehyde and prepared for frozen sections of 5-µm thickness as described above. After terminal dUTP-mediated nick-end-labeling (TUNEL) staining and nuclei staining with Hoechst 33342, tumor sections were examined by a fluorescence microscope (Leica DMI 4000B, Germany) to detect cell apoptosis. The apoptosis percentage of tumor cells from six randomly-assigned regions in each group (n = 6) was quantified by the ImageJ software. Tumor growth inhibition rates (*TGIR*) based on tumor size (*TGIR*_*V*_) and tumor weight (*TGIR*_*W*_) were calculated by the following formulas, respectively: ${{TGIR}}_{V}=\frac{{V}_{c}-{V}_{t}}{{V}_{c}}$; ${{TGIR}}_{V}=\frac{{W}_{c}-{W}_{t}}{{W}_{c}}$. In these formulas, *V*_*c*_ and *V*_*t*_ represented the tumor volume in the control group and that in the treatment group, respectively; *W*_*c*_ and *W*_*t*_ represented the tumor weight in the control group and that in the treatment group, respectively.

### Statistical analysis

Statistical differences were evaluated with an unpaired Student’s *t*-test for two groups’ comparison and one-way analysis of variance (ANOVA) for multiple-group comparison. Data are expressed as mean ± standard deviation (SD), and *P* values < 0.05 were considered statistically different.

## Results and Discussion

When the A549 tumor xenograft reached 4 to 5 mm in diameter, celecoxib treatment was initiated. After the two-week celecoxib treatment at the celecoxib dose of 200 mg/kg/day, there were no significant differences between the two groups in tumor size or body weight of tumor xenograft-bearing mouse models (Fig. S1A). In addition, no obvious necrosis or inflammation occurred in the major organs of the two groups and there were no significant differences between the control group and celecoxib group (Fig. S1B). These results indicated no severe adverse effects were associated with celecoxib treatment in the present study, which might be due to that celecoxib was a clinically widely used drug with a good safety and the dose of celecoxib was 200 mg/kg/d for two weeks in the present study, much less than 330 mg/kg/d for six weeks in previous report^32^.

As previously reported, the inhibition effect of celecoxib treatment on tumor mass growth was closely associated with the dosage form^22^, the administration time or route^19, 33^, tumor types^21^, etc. For instance, when celecoxib treatment started on the day of tumor cell implantation^19^, oral celecoxib treatment (100 mg/kg/day) for one week could suppress tumor growth significantly in Lewis lung carcinoma-bearing mouse models, reflecting the crucial role of COX-2-associated inflammation in initial tumor growth^34^. However, as tumor xenografts grow, tumor growth might not depend solely on the COX-2-associated signaling pathway and much higher doses of celecoxib might be needed to interrupt tumor growth. Therefore, oral celecoxib treatment at a dosage of 200 mg/kg/day for two weeks might only normalize the tumor microenvironment but might exert almost no effect on tumor mass growth in the present study.

**Figure 2:**
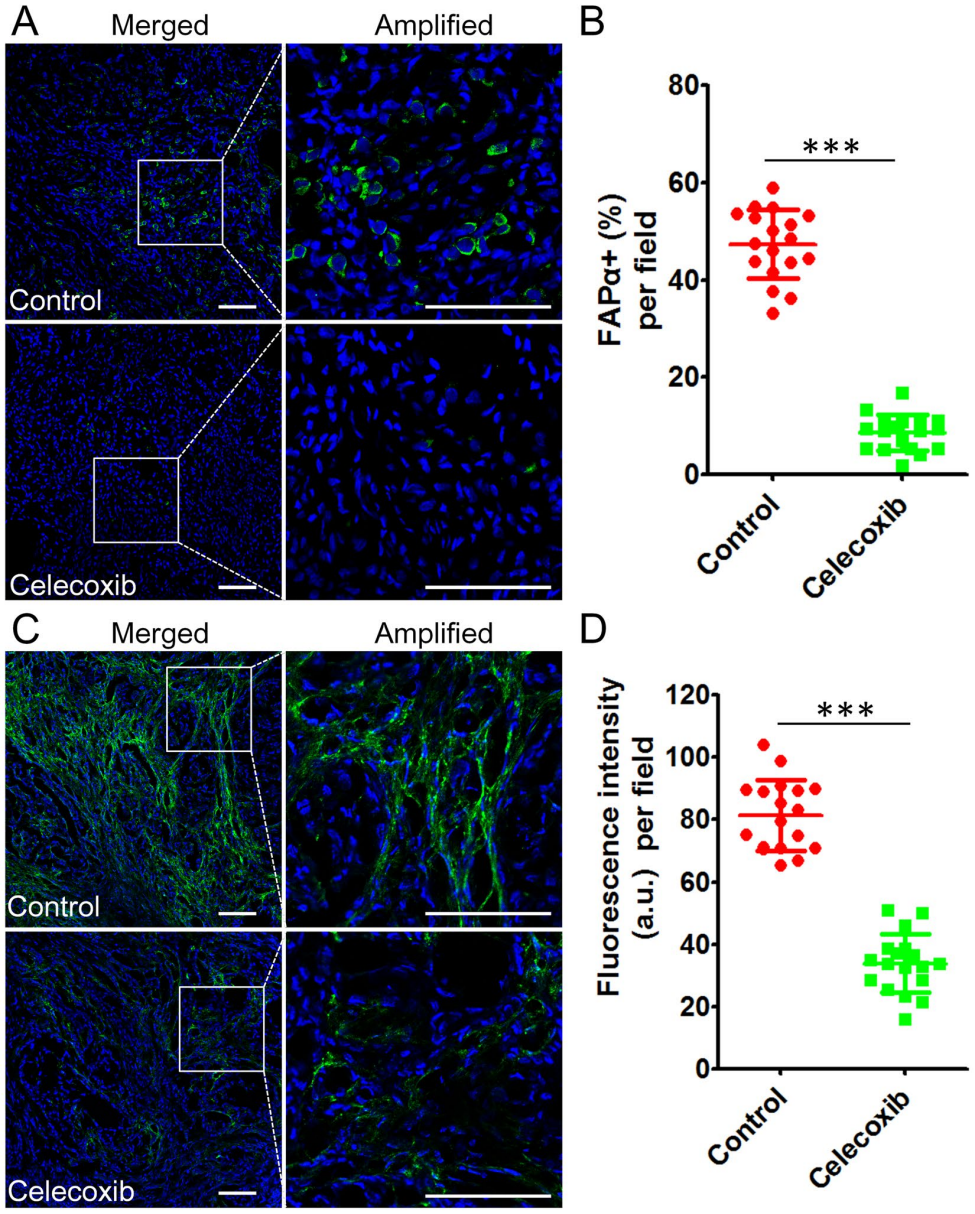
Oral celecoxib treatment reduced TAF and disrupted tumor fibronectin bundles demonstrated by immunofluorescence staining. (**A**) TAF expression (green) in the tumor tissues with or without celecoxib treatment and (**B**) the corresponding percentage of TAF (FAPα+) in the tumor tissue cells per field (n = 18). The TAF was labeled by FAPα antibody. (**C**) The fibronectin expression (green) in the tumor tissue with or without celecoxib treatment and (**D**) the corresponding fluorescence intensity of fibronectin per field (n = 18). The oral dose of celecoxib was 200 mg/kg/day for 14 days. The bar indicates 100 μm, and the nuclei were stained with Hoechst 33342 (blue). ***P < 0.0001 vs. the control group.

As the principal cellular component of the tumor microenvironment, TAF is the main source of ECM. Mechanical vascular compression from the components of the tumor microenvironment, including TAF and ECM, combined with the abnormal structure of tumor vessels contributes to heterogeneous tumor perfusion^4, 8^. In addition, both TAF and ECM could effectively hinder nanotherapeutics from freely reaching tumor cells^35^. In the present study, the effect of celecoxib treatment on the tumor microenvironment, including TAF, ECM, tumor vessels, and tumor perfusion was investigated by immuno-fluorescence staining. The results showed that the percentage of FAPα-positive TAF in tumor tissues was markedly reduced by celecoxib treatment, from 47.4 ± 7.0% in the control group to 8.4 ± 3.2% in the celecoxib group (Fig. 2A,B). The significant TAF depletion might be attributed to four reasons as follows. First, TAF mainly comprises tumor stromal cells, and most of these were recruited from the bone marrow. Celecoxib inhibited the CXCL12/CXCR4 axis, which might play a crucial role in tumor stromal formation and angiogenesis under the control of prostaglandins and attenuated TAF recruitment from the bone marrow^19^. Second, celecoxib could suppress fibroblast proliferation and activation stimulated by FGF-2 and transforming growth factor beta-1 (TGF-beta1) by inhibiting ERK1/2 phosphorylation^36^. Third, celecoxib might induce G1-S cell cycle arrest and apoptosis of TAF similar to leukemia cells^37^. Finally, TAF might be reprogrammed into normal fibroblasts by celecoxib like M2 macrophages were reeducated to M1 macrophages^38^.

As one of the most important components of ECM, fibronectin has been used as the therapeutic target in some ECM-targeting tumor therapy^26, 39^, and it was selected as the ECM marker in the present study^25, 40^. As shown in Fig. 2C, fibronectin bundles were disrupted and became fractured after celecoxib treatment, while they were compact and regular in the control group. Additionally, fibronectin expression in tumor tissues significantly decreased to 41.5% in the celecoxib treatment group as compared with that in the control group (Fig. 2D), probably due to the TAF reduction and the suppression of TAF-producing ECM^19, 36^. These results indicated that celecoxib treatment markedly reduced the expression of TAF and ECM in tumor tissues, which might help depress the tumor vessels and benefit the tumor perfusion and nanotherapeutics penetration in tumor tissues^6, 8^.

**Figure 3:**
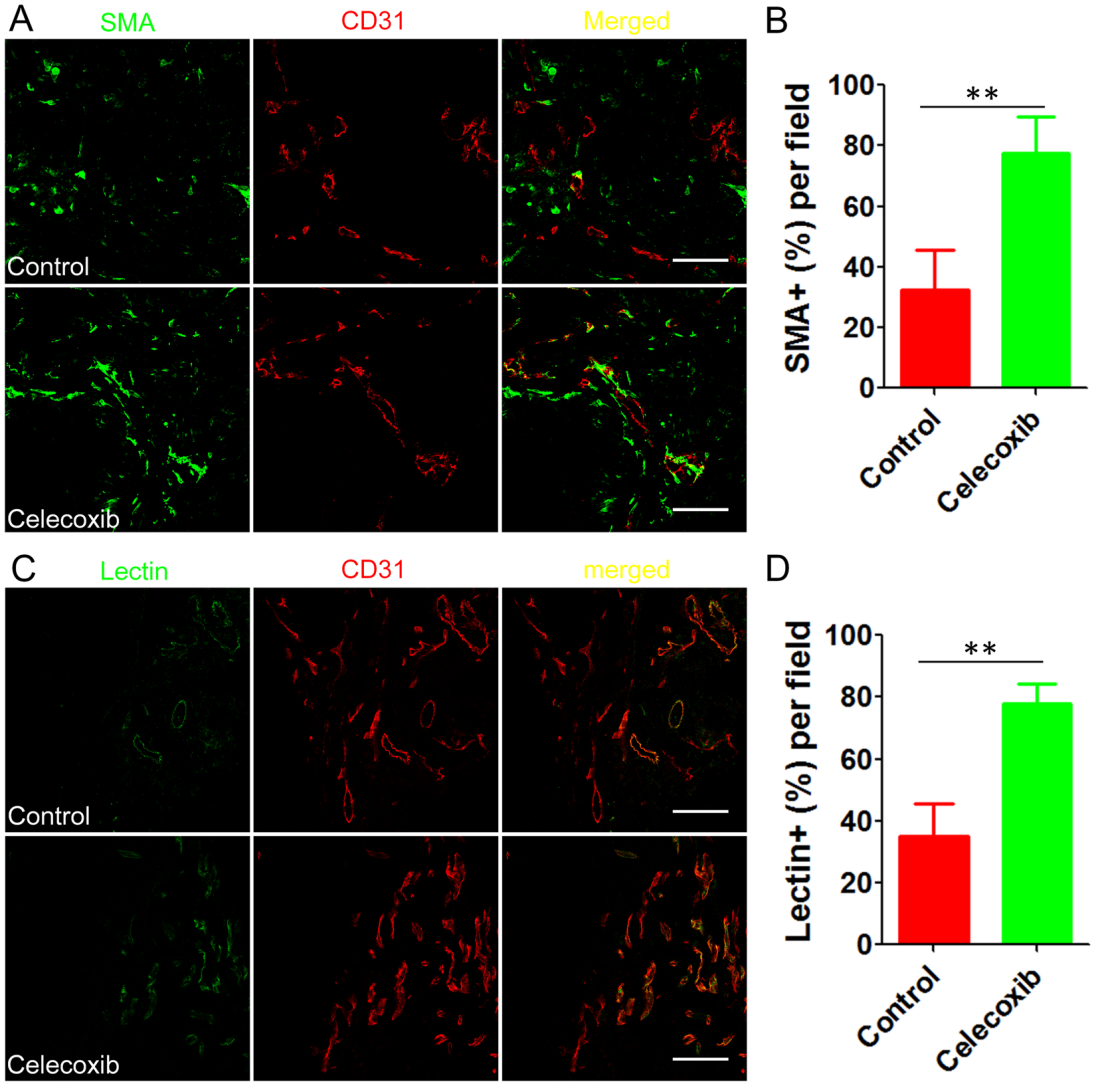
Oral celecoxib treatment normalized tumor vessels and improved tumor perfusion. (**A**) The pericyte (green) coverage on tumor vessels (red) in the tumor tissues with or without celecoxib treatment and (**B**) the corresponding percentage of the tumor vessels covered by pericytes (n = 18). SMA antibody was used to label the pericytes. (**C**) The tumor perfusion after celecoxib treatment was indicated by lectin labeling (green) and (**D**) the corresponding percentage of the perfused tumor vessels in all tumor vessels (red) (n = 18). Lectin and CD31 was used to label perfused vessels and tumor vessels (including well perfused and non-perfused vessels), respectively. The oral dose of celecoxib was 200 mg/kg/day for 14 days. The bar indicates 100 μm. **P < 0.001 vs. the control group.

Microvessel density in tumor stroma was assessed as a parameter of tumor-associated angiogenesis. Celecoxib treatment significantly decreased the microvessel density from 234 ± 43/mm^2^ in the control group to 186 ± 25/mm^2^ in the celecoxib group, exhibiting evidence of less angiogenesis, which agreed well with previous report^19^. The coverage of pericytes on endothelial cells was used as an indicator of tumor vessel normalization^27^. Celecoxib treatment significantly increased the percentage of tumor vessels covered by pericytes from 32.4 ± 13.4% in the control group to 77.6 ± 12.0% in the celecoxib group (Fig. 3A,B), indicating that celecoxib could normalize A549 tumor vessels. Further scanning electron microscopy of tumor vessels might display the normalized vessel structure after celecoxib treatment^41^. The lectin-labeling experiment revealed that celecoxib treatment significantly increased the percentage of functional blood vessels from 35.1 ± 4.5% in the control group to 77.8 ± 3.8% in the celecoxib group. The perfusion improvement in tumor tissues might be attributed to TAF reduction, ECM disruption, tumor vessels normalization by celecoxib treatment. Altogether, these results verified that celecoxib treatment comprehensively normalized the tumor microenvironment, which might be related to COX-2 inhibition and prostaglandin reduction in tumors^19, 42^.

Previously, tumor microenvironment modifiers, including bacterial collagenase, relaxin, and matrix metalloproteinase^11, 43, 44^, were reported to disrupt the collagen or proteoglycan network in tumor tissues and enhance tumor drug delivery to various degrees, but with the potential to increase side effects to normal tissues or the risk of tumor metastasis^7^. In comparison, the clinically widely used drug celecoxib could not only normalize tumor vessels to reduce the shedding of tumor cells into the circulatory system, which was regarded as a prerequisite for tumor metastasis^3^, but also inhibits COX-2-associated inflammatory conditions in the potential metastasis site to reduce tumor metastasis occurrence^34^. Thus, celecoxib might be safely and effectively used in tumor microenvironment normalization.

**Figure 4:**
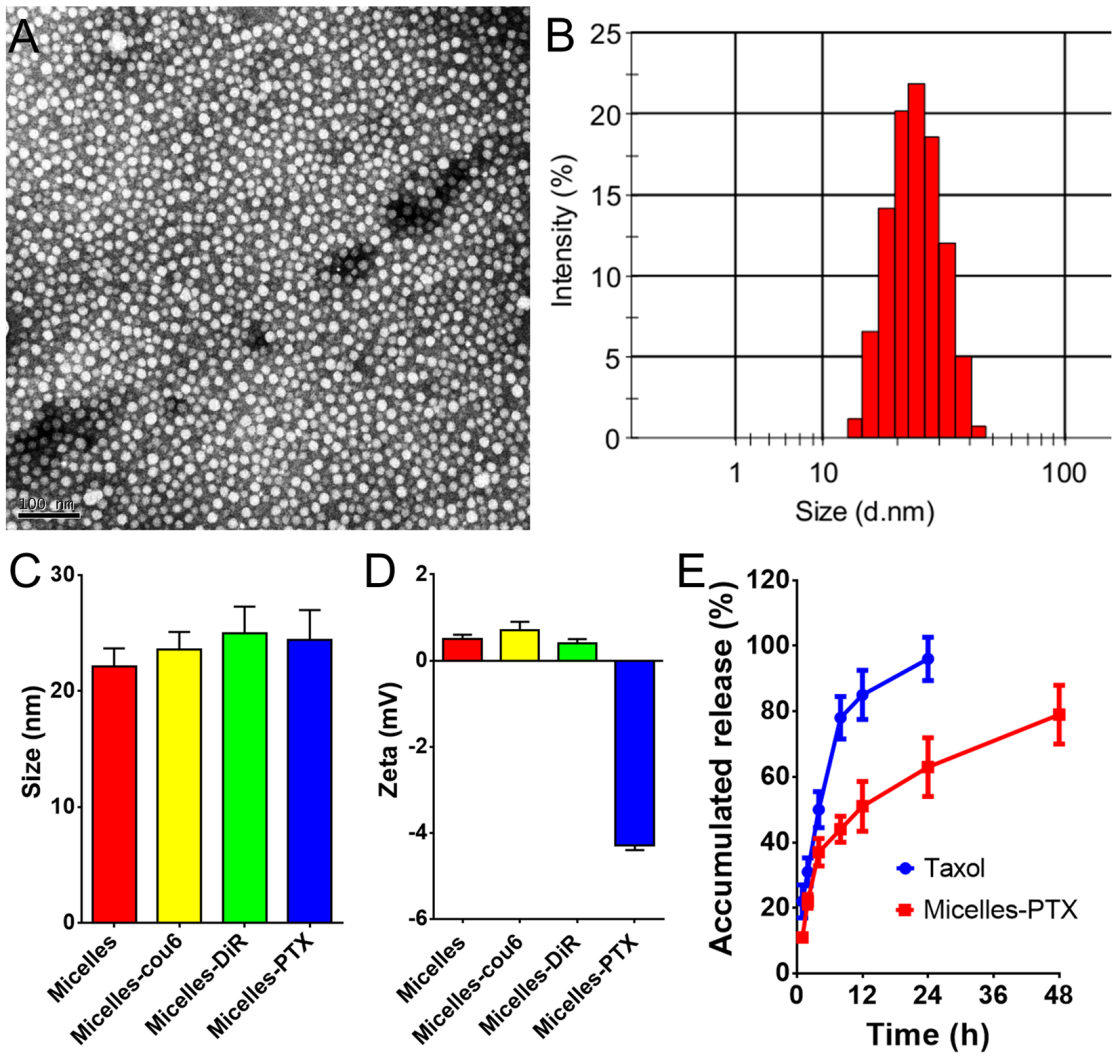
Characterization of small micelles. (**A**) TEM image of micelles negatively stained by 2% phosphotungstic acid. (**B**) Particle size distribution of micelles analyzed by DLS. (**C**) Particle size and (**D**) zeta potential of drug-loaded micelles. (**E**) PTX-releasing profiles from Taxol, Micelles-PTX in PBS (0.01 M, pH = 7.4) with 0.5% Tween-80 at 37 °C (n = 4).

Currently, nanotherapeutics based on food and drug administration (FDA)-approved polymer materials, such as polyethylene glycol (PEG), poly (D, L-lactide-co-glycolide) (PLGA), polylactic acid (PLA), etc. have aroused increasing interest in the field of tumor drug delivery due to their advantages, including low cost, favorable biocompatibility, good hydrophilicity, and simplicity of nanotherapeutics synthesis^45–47^. In particular, PTX micelles free of Cremophor EL with fewer side effects have already been clinically approved in South Korea, and are now in Phase II clinical trials in the US^48^. However, the advanced design of nanotherapeutics alone could not conquer those barriers presented by the complex tumor microenvironment, and normalization of the tumor microenvironment appeared to be extremely important for improving tumor nanomedicine delivery^49^. To investigate the effect of tumor microenvironment normalization by celecoxib treatment on the *in vivo* delivery of nanotherapeutics, Micelles-PTX was selected as the model drug in the present study. Blank micelles were developed by a thin-film hydration method and the TEM image showed that micelles were of a regular size and smooth surface (Fig. 4A). The mean diameter of blank micelles was 22.1 ± 1.5 nm (Fig. 4B) with a narrow distribution (PDI 0.06), which was in good agreement with the TEM image, and their zeta potential was 0.5 ± 0.1 mV. Fluorescence dye labeling did not significantly change the size and the zeta potential of micelles (Fig. 4C,D). Encapsulation of chemotherapeutic PTX into micelles did not significantly change the size of micelles, but decreased the zeta potential (Fig. 4C,D), which agreed with our previous report^50^. The drug loading of PTX in micelles was 23.1 ± 2.6%. The *in vitro* PTX release profile showed that the PTX released from Micelles-PTX was slower than that from Taxol (Fig. 4E), which agreed well with the previously reported results^51^.

**Figure 5:**
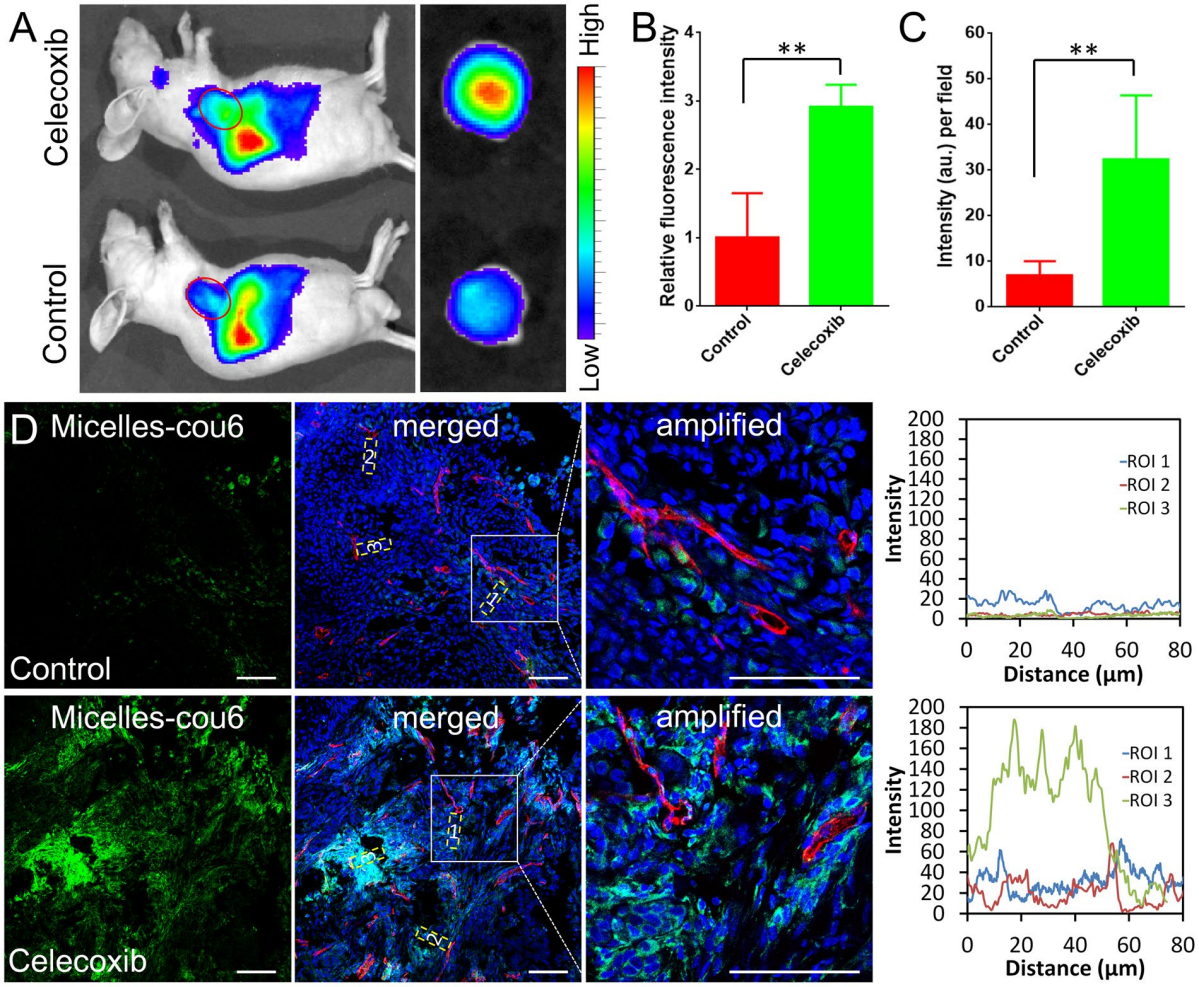
The effect of celecoxib treatment on the tumor delivery of micelles. (**A**) *In vivo* imaging of A549 xenograft-bearing mice (left) and e*x vivo* imaging of tumor xenografts (right) treated with or without celecoxib 24 h post-DiR-labeled micelles administration. (**B**) Relative fluorescence intensity per gram tissue in the homogenate of tumor xenografts (n = 4). (**C**) The fluorescence intensity of coumarin-6-labeled micelles per field in the tumor slices treated with or without celecoxib (n = 4). (**D**) *In vivo* distribution and penetration profiles of coumarin-6-labeled micelles in tumor tissues treated with or without celecoxib displayed by immunofluorescence staining. Right panels show the fluorescence intensity profiles of micelles from the nearest blood vessels to the tumor tissue in the selected regions of interest (ROI, indicated by dashed yellow rectangles). The dosage regimen of celecoxib was 200 mg/kg/day by gavage once a day for 14 days. **P < 0.01 compared with the control group. Blue: nuclei. Green: coumarin-6-labeled micelles. Red: CD31-labeled tumor vessels. The bar indicates 100 μm.

The concomitant effect of celecoxib on nanotherapeutics delivery was investigated by an *in vivo* imaging and distribution experiment. The results of *in vivo* imaging showed that 22-nm micelles could accumulate more selectively in A549 xenografts as compared with the control group (Fig. 5A), with the fluorescence signal intensity significantly increased by 2.9-fold (Fig. 5B). Furthermore, celecoxib treatment did not influence the micelle distribution in normal organs, which further verified its safety superiority (Fig. S2). By contrast, celecoxib treatment decreased the tumor distribution of 101-nm nanoparticles to some extent, with the fluorescence signal that measured approximately 56.7% of that of control group (Fig. S3). The results might be attributed to the vascular normalization by celecoxib treatment. It increased the coverage of pericytes on endothelial cells and reduced the vascular pore size, preferentially benefiting the delivery of free drugs or smaller nanoparticles (diameter, 10 to 40 nm) while hampering the delivery of relatively larger nanoparticles^52, 53^. The size-dependent drug delivery required us to optimize the tumor microenvironment and nanotherapeutics simultaneously to boost the therapeutic benefits of nanotherapeutics for tumor treatment^49^.

The overall high accumulation of nanotherapeutics in tumor tissues alone did not always guarantee optimal therapeutic efficacy, and a homogeneous distribution pattern might be more crucial for the final performance of nanotherapeutics^54, 55^. Consequently, a green fluorescence tracker, coumarin-6 with inertness to leak, was utilized to label micelles to track their distribution pattern *in vivo*. The results demonstrated that more micelles were accumulated in tumor tissue in celecoxib group than those in the control group (Fig. 5C,D). Moreover, micelles were mainly located in the perivascular region in the control group. By contrast, in the celecoxib-treated group, micelles could penetrate more deeply into the tumor parenchyma and distribute more homogeneously in the tumor tissues (Fig. 5D); with the potential to reach more tumor cells and exert a better therapeutic effect. The main reasons for the improved accumulation and distribution of micelles in tumors after celecoxib treatment might be the following factors: Celecoxib treatment reduced TAF expression and disrupted ECM, which depressed tumor vessels, improved tumor perfusion, and potentially reduced the resistance of micelle penetration within the tumor. Celecoxib treatment also normalized tumor vessels, which directly ameliorated tumor perfusion and increased the global distribution of small-size micelles in tumors. Therefore, the favorable accumulation and distribution pattern of micelles facilitated by celecoxib treatment predicted a potent tumor growth inhibition effect when the therapeutics was loaded.

**Figure 6:**
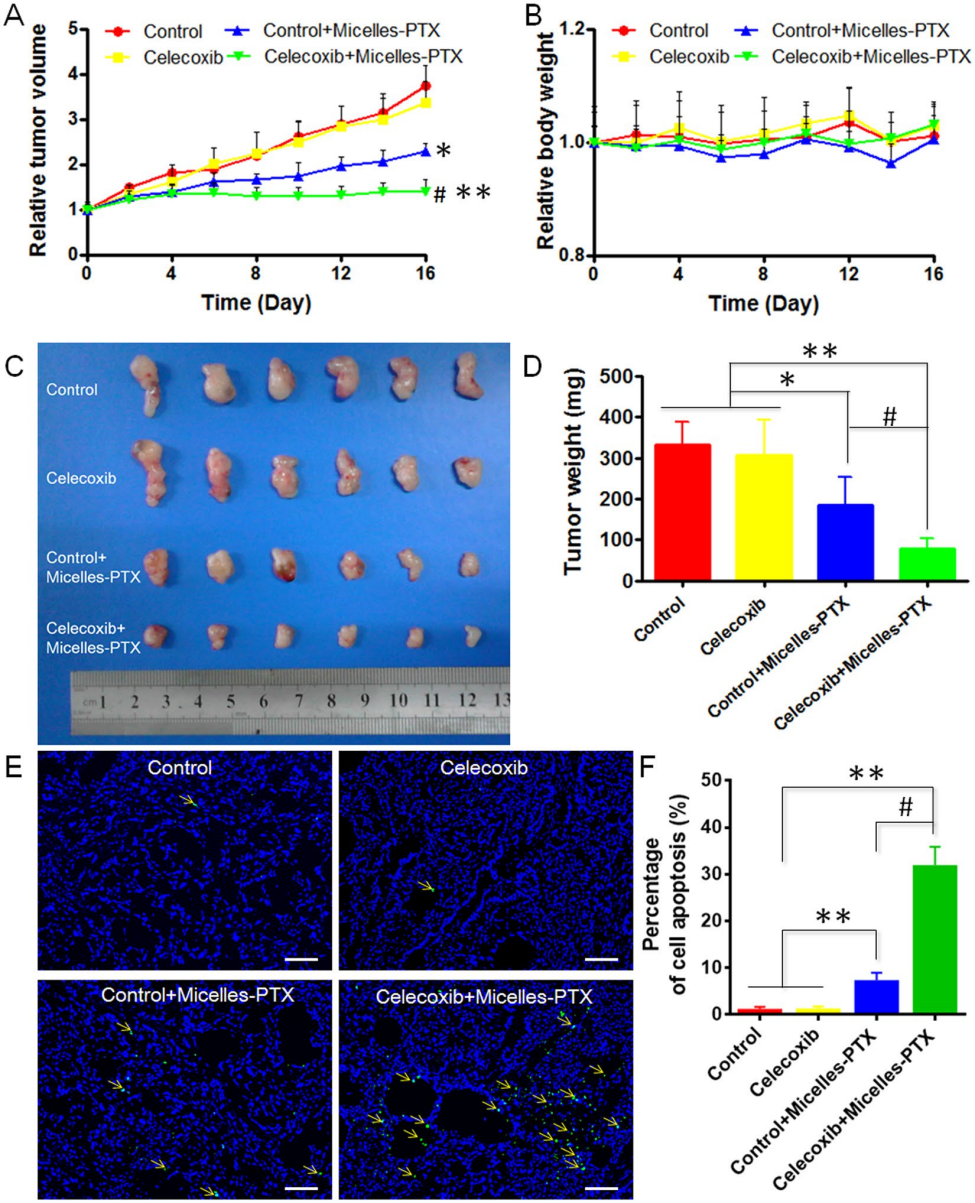
Oral celecoxib treatment improved the chemotherapeutic efficacy of micelles-PTX. Mice bearing A549 tumors were orally administered either celecoxib or not for two weeks and then subjected to i.v. injection of micelles-PTX every 3 d for five consecutive injections (Day 0, 3, 6, 9, 12) at a dosage of PTX 6 mg/kg. On day 16, mouse models in each group (n = 6) were sacrificed, and tumor xenografts were collected and weighed. (**A**) Tumor volume curves and (**B**) body weight curves of mouse models during the whole experiment. (**C**) Images of tumor xenografts and (**D**) tumor weights were obtained at the study endpoint. (**E**) TUNEL staining of tumor slices was performed (Blue: nuclei. Green: cell apoptosis indicated by yellow arrows. The bar indicates 100 μm) and (**F**) the corresponding semiquantitative percentage of cell apoptosis was displayed in the histogram. *P < 0.05, **P < 0.01 vs. the control or celecoxib group; #*P* < 0.01 vs. the micelles-PTX group.

Finally, we tested the growth inhibition effects of Micelles-PTX plus celecoxib pretreatment on size-matched A549 xenografts. As shown in Fig. 6A, the growth curves of the A549 xenografts confirmed the findings of the *in vivo* accumulation and distribution of micelles experiments. Poor tumor growth inhibition was observed in the celecoxib group and modest tumor growth inhibition was achieved in the control plus Micelles-PTX group. As expected the most prominent tumor shrinkage was found in the celecoxib plus Micelles-PTX group. The *TGIRv* and *TGIRw* for the celecoxib plus Micelles-PTX group were 71.4% and 76.8% (Fig. 6C,D), respectively, which were significantly higher than those for the control plus Micelles-PTX group (39.1% and 44.8%, respectively). Consistent with the tumor growth experiment, tumor histology assessment by TUNEL assay revealed that celecoxib treatment could markedly enhance tumor cell apoptosis induced by Micelles-PTX treatment (Fig. 6E,F). As a classical representative of clinical non-steroidal anti-inflammatory drugs (NSAIDs) with no selectivity between COX-1 and COX-2, aspirin was reported to improve small-molecule chemotherapeutics delivery for tumor therapy^56, 57^ in both animal models and clinics, and the related mechanism might be the mTOR signaling pathway inhibition^53, 58^. However, aspirin was always associated with COX-1-related adverse effects including the promotion of bleeding and the induction of gastrointestinal complications^17^. Additionally, the mTOR signaling inhibition only normalized tumor vessels but exert little effect on tumor matrix or tumor stroma cells. As a comparison, as a selective COX-2 inhibitor, celecoxib was not only able to avoid COX-1-associated side effects, but also exerted a more comprehensive effect on normalizing the tumor microenvironment than aspirin, and improved small nanotherapeutics delivery for tumor treatment, showing more promising prospects of clinical translation than aspirin.

## Conclusion

The present study represents the first time that a selective COX-2 inhibitor, celecoxib, was explored to help improve tumor drug delivery. Celecoxib could comprehensively modify the tumor microenvironment, including tumor-associated fibroblast reduction, fibronectin bundle disruption, tumor vessel normalization and tumor perfusion improvement. Furthermore, it also increased *in vivo* delivery of micelles and improved therapeutic benefits of PTX-loaded micelles. As celecoxib is now safely and widely used in clinics, our findings may have great potential in clinics to improve solid tumor treatment.

## Electronic supplementary material

Celecoxib supporting materials
