# Supplementary material for: Celecoxib normalizes the tumor microenvironment and enhances small nanotherapeutics delivery to A549 tumors in nude mice
Source: Sci Rep. 2017 Aug 30;7:10071. doi: 10.1038/s41598-017-09520-7 (PMC5577220; doi:10.1038/s41598-017-09520-7)
Supplement: Supplementary file 1 — Celecoxib supporting materials [file 41598_2017_9520_MOESM1_ESM.pdf]

Supplemental information

Celecoxib normalizes the tumor microenvironment and enhances small nanotherapeutics delivery to A549 tumors in nude mice

Bo Zhang, Kai Jin, Ting Jiang, Lanting Wang, Shun Shen, Zimiao Luo, Yanyan Tuo, Xianping Liu, Yu Hu*, Zhiqing Pang*

* Corresponding author:

Yu Hu, dr_huyu@126.com

Zhiqing Pang, zqpang@fudan.edu.cn

Methods

Nanoparticles preparation and characterization

DiR-labeled nanoparticles (NP-DiR) were prepared using the emulsion/solvent evaporation technique as previously reported [1,2]. Briefly, 24 mg of MPEG-PLA (Mw 33000 Da) and 200 μg of DiR were dissolved in 1 ml of dichloromethane and added to 5 ml of 0.6% sodium cholate aqueous solution, and then was sonicated (200 W, 5 s for 15 times) on ice by a probe sonicator (Scientz Biotechnology Co. Ltd., China). After evaporating dichloromethane with a ZX-98 rotary evaporator (Shanghai Institute of Organic Chemistry, China), NP-DiR was collected by concentrating with a TJ-25 centrifuge (Beckman Counter, USA). The particle size was 101.5±6.8 nm (n=4) and the zeta potential was -9.0±0.9 mV (n=4), which were analyzed using a Malvern Nano ZS (Malvern Instruments, UK). The drug loading of DiR in nanoparticles was 0.65±0.03% (n=4) which was determined by the HPLC method [2].

In vivo imaging of NP-DiR in A549 tumor-bearing mouse models

At the end of two-week celecoxib treatment, A549 xenograft-bearing mouse models were injected with NP-DiR at the DiR dose 0.5 mg/kg. Twenty four hours later, the *in vivo* fluorescence imaging of mouse models was performed with the *In Vivo* IVIS spectrum imaging system (PerkinElmer, USA). Mouse models were then sacrificed followed by heart perfusion with saline. Tumors were collected and subjected to *ex vivo* imaging with the *In Vivo* IVIS spectrum imaging system (PerkinElmer, USA). Additionally, the tumors were carefully weighed and then homogenized in 0.01 M PBS (pH=7.4). The fluorescence intensity of each sample was analyzed by a Tecan Infinite M200 Pro Multiplate Reader (Switzerland) with the excitation wave length of 748 nm and emission wave length of 780 nm.

Results

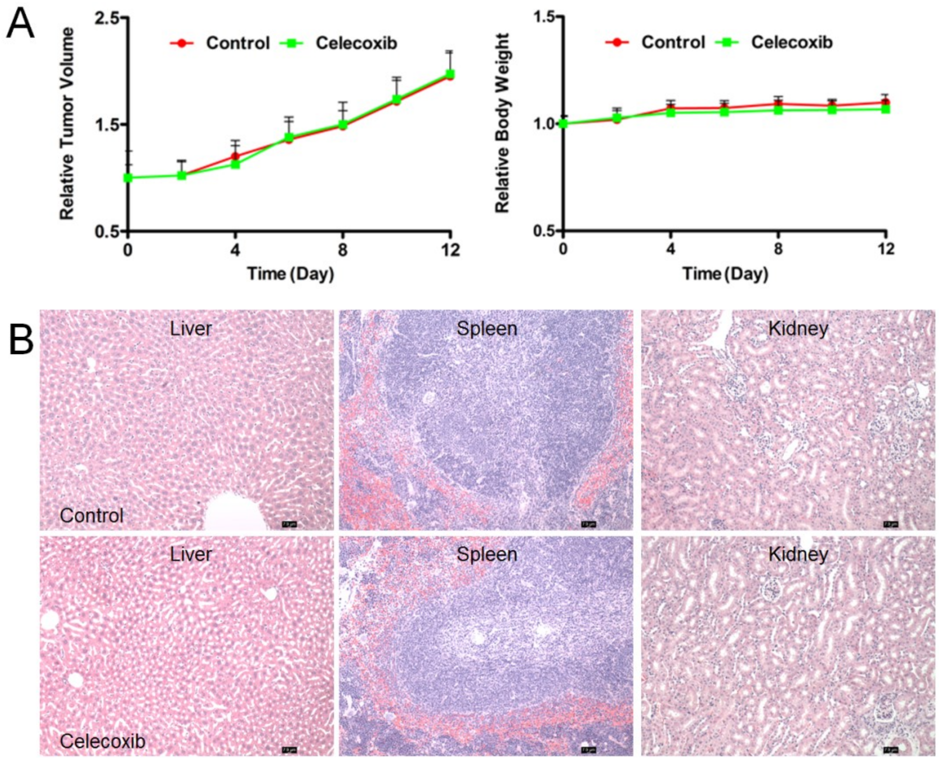

Fig S 1 (A) Effect of celecoxib treatment on tumor size (left) and body weight (right) of A549

tumor xenograft-bearing mouse models (n=6). (B) H&E staining of major organs from tumor xenograft-bearing mouse models treated with celecoxib (bar=7.9 μm). The dosage regimen of celecoxib was 200 mg/kg/day by gavage once a day for 14 days.

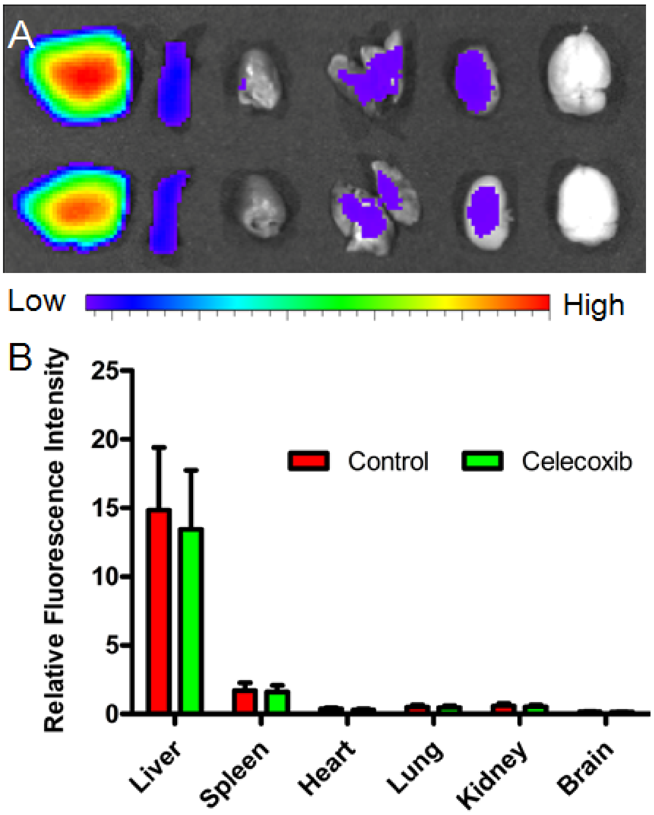

Fig S 2 (A) *Ex vivo* imaging of major organs (from left to right: liver, spleen, heart, lung, kidney and brain) collected from mice models treated with celecoxib (lower row) or without celecoxib (upper row) by the *in vivo* imaging system and (B) the corresponding quantitative results of these organs 24 h post DiR-labeled micelles administration (n=4). The gavage dosage of celecoxib was 200 mg/kg/d for 14 days.

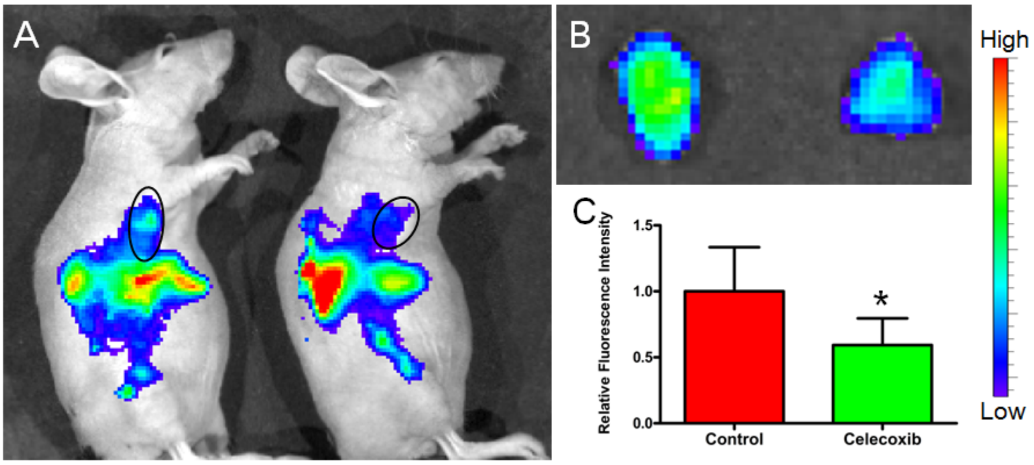

Fig S 3 (A) *In vivo* imaging of A549 xenograft-bearing mice treated with celecoxib (right) or without celecoxib (left) 24 h post DiR-NP administration. (B) *Ex vivo* imaging of tumors 24 h post DiR-NP administration (Left: control; Right: celecoxib) and (C) the corresponding quantitative results (n=4). The dosage regimen of celecoxib was 200 mg/kg by gavage once a day for 14 days. *$P<0.05$ compared with the control group.

References

1. Zhang, B. *et al.* UPA-sensitive ACPP-conjugated nanoparticles for multi-targeting therapy of brain glioma. *Biomaterials* **36**, 98-109, (2015).
2. Zhang, B. *et al.* LDLR-mediated peptide-22-conjugated nanoparticles for dual-targeting therapy of brain glioma. *Biomaterials* **34**, 9171-9182, (2013).
